# A randomized controlled trial of adjunctive iTBS targeting the dorsomedial prefrontal cortex for anhedonia in schizophrenia: rationale and protocol design

**DOI:** 10.3389/fpsyt.2025.1706553

**Published:** 2025-11-24

**Authors:** Hao Wu, Mei Yu, Yuke He, Wenzhong Liu, Yang Fan, Wei Xu, Han Den, Yun Long, Bo Liu, Kezhi Liu, Youguo Tan

**Affiliations:** 1 The Affiliated Hospital, Southwest Medical University, Luzhou, China; 2 The Zigong Affiliated Hospital, Southwest Medical University, Zigong, China; 3 Research Center for Psychiatry, Zigong Institute of Brain Science, Zigong, China

**Keywords:** schizophrenia, anhedonia, rTMS, iTBS, dmPFC, fNIRS, cognitive functions

## Abstract

**Introduction:**

Anhedonia, a core negative symptom of schizophrenia, refers to the inability to experience pleasure from normally enjoyable activities. It significantly worsens prognosis and remains poorly addressed by current treatments. In recent years, repetitive transcranial magnetic stimulation (rTMS) has shown potential in treating anhedonia in depression and other disorders; however, its application to anhedonia in schizophrenia remains underexplored. This study aimed to evaluate the efficacy of rTMS targeting the dorsomedial prefrontal cortex (DMPFC) on anhedonia in patients with schizophrenia, and to explore its correlation with cognitive function and prefrontal activity.

**Methods:**

The present study is a randomized controlled trial that will include 82 patients with schizophrenia who meet DSM-5 diagnostic criteria and have a score of ≥12 on the Snaith-Hamilton Pleasure Scale (SHAPS). Eligible participants will be randomly allocated in a 1:1 ratio to either active or sham rTMS stimulation groups. The active group will receive a high-frequency rTMS intervention using an intermittent theta-burst stimulation (iTBS) protocol (20 trains x 10 pulses at 5 Hz, with an intensity of 90% of resting motor threshold) administered twice daily for 15 days. The sham group will receive a sham-stimulation regimen with the same parameters. All participants will be assessed using both the SHAPS and the Scale for the Assessment of Negative Symptoms (SANS) at three time points: baseline (T0), immediately post-treatment (T1), and 4-week follow-up (T2). In addition, the Wisconsin Card Sorting Test (WCST) and the dynamics of prefrontal oxygenated hemoglobin (Δoxy-Hb) monitored by functional near-infrared spectroscopy (fNIRS), will be assessed at T0, T1, and T2.

**Ethics and dissemination:**

The trial protocol complies with the principles of the Declaration of Helsinki and has been approved by the Ethics Committee of Zigong Mental Health Center (approval number: 20250801). The findings of this trial will be published and made publicly accessible in a peer-reviewed journal.

**Discussion:**

If the iTBS protocols targeting the DMPFC lead to positive changes in anhedonia symptoms in patients with schizophrenia, this would provide a new intervention strategy for negative symptoms. Furthermore, leveraging neuroimaging evidence from such studies could help optimize rTMS target selection and overall treatment strategies.

Trial registration: ChiCTR2500107965.

## 1 Introduction

Schizophrenia is a severe mental disorder, with a 12-month prevalence of approximately 0.6% among Chinese adults—rising to as high as 1.3% in those aged 18 to 34 years—and a lifetime prevalence of about 1% (1). It is characterized primarily by positive symptoms, negative symptoms, and cognitive impairments. Among these, negative symptoms are broadly defined as reductions in motivation and interest (e.g., diminished volition, anhedonia, social withdrawal) or in expressive functioning (e.g., affective flattening, poverty of speech) (2). Research has shown that symptoms involving low motivation and diminished interest are associated with poorer outcomes than those involving reduced emotional expression, suggesting they may represent a more severe aspect of schizophrenia (3).

Anhedonia refers to the inability to experience pleasure from activities or events that are typically enjoyable. Both general and domain-specific anhedonia are significantly more severe in individuals with schizophrenia compared to the general population (4). Anhedonia is associated with multiple adverse consequences, including reduced educational attainment, higher unemployment rates, increased mental health service utilization (5), and greater suicide risk (6). Evidence suggests that individuals with schizophrenia who exhibit anhedonia respond less favorably to treatment than those without this symptom (3). As such, the elucidation of its neurobiological basis and the development of effective treatments for anhedonia are essential for advancing treatment and improving long-term outcomes in schizophrenia (7).

Non-invasive brain stimulation (NIBS), including repetitive transcranial magnetic stimulation (rTMS) and transcranial direct current stimulation (tDCS), has emerged as a potential treatment modality for negative symptoms in schizophrenia (8, 9). A meta-analysis by Chen et al. on studies of NIBS for negative symptoms in schizophrenia found that NIBS overall had a moderate effect size on negative symptoms (SMD = -0.54), with particularly significant effects in the dimensions of anhedonia (SMD =-0.63) and avolition (SMD = -0.47) (10). Lisoni et al. demonstrated that bilateral unbalanced prefrontal tDCS clearly improved the avolition and apathy domains (11). However, not all NIBS studies have reported positive results. For instance, Kos et al. found that neither rTMS nor tDCS targeting the right DLPFC significantly improved apathy or negative symptoms in patients with schizophrenia. The authors suggested that this might be related to factors such as the stimulation target and the relatively low total number of pulses (12).

Accumulating evidence suggests that dysregulation of the brain’s reward circuitry plays a central role in the pathophysiology of anhedonia in schizophrenia (13). The dorsomedial prefrontal cortex (DMPFC) is closely associated with the midbrain-limbic dopaminergic system, which constitutes the brain’s reward circuitry. It plays an important role in reward processing through its interactions with this dopaminergic system (14). A recent study found that rTMS applied to the DMPFC can improve depressive symptoms in depressed patients by improving the connectivity of reward circuits, and specifically helps to alleviate anhedonia (15). Emerging clinical evidence—including case reports or case series in major depressive disorder (MDD) (16) and bipolar disorder (17), as well as studies in post-traumatic stress disorder (PTSD) (18) and eating disorders (19)—suggests that rTMS targeting DMPFC (DMPFC-rTMS) is safe, tolerable, and potentially effective. Additionally, preliminary findings also indicate that DMPFC-rTMS may ameliorate negative symptoms in schizophrenia (20). Thus, DMPFC may be a promising target for rTMS to improve anhedonia in schizophrenia.

Intermittent theta burst stimulation (iTBS) is a specific rTMS protocol consisting of 20 trains. Each train includes a 2-second stimulation period followed by an 8-second inter-train interval. The iTBS pattern delivers clusters of three pulses at 50 Hz, repeated at a 5 Hz (theta) frequency, for a total of 600 pulses per session (21, 22). This modality has an excitatory effect, and its efficacy in ameliorating anhedonia in depressed patients is comparable to or even exceeds that of traditional modalities (22). In recent years, multiple systematic reviews and meta-analyses have provided further support for the potential of iTBS in improving symptoms of schizophrenia, particularly negative symptoms. A systematic review by Tan et al., which included 12 randomized controlled trials (involving a total of 637 patients), found that iTBS had a moderate effect size on negative symptoms (SMD = 0.59, p = 0.03) (23). Additionally, the study did not find any significant effects of iTBS on positive or depressive symptoms, suggesting that its effects may be symptom-specific. Another meta-analysis conducted by Wen et al. compared the efficacy of iTBS with conventional rTMS, showing that iTBS was superior to rTMS in improving negative symptoms (SMD = -0.43, p = 0.04), while no significant differences were observed in overall psychopathology, positive symptoms, or general symptoms (24). A more comprehensive network meta-analysis further confirmed that, among all theta-burst stimulation (TBS) modalities, only iTBS was significantly associated with improvements in negative symptoms (SMD = -0.89), overall symptoms, depression, anxiety, and cognitive function, with good tolerability (25). Thus, DMPFC-iTBS may be a promising approach to improve anhedonia in schizophrenia.

Furthermore, previous studies have demonstrated a significant correlation between cognitive function and anhedonia (7, 26). Functional near-infrared spectroscopy (fNIRS) is a relatively non-invasive, well-tolerated, cost-effective optical neuroimaging tool. It can measure task-related changes in oxygenated hemoglobin and deoxygenated hemoglobin in the cerebral cortex (27), thereby revealing alterations in brain function, making it highly valuable for studying cognitive functions and brain activity (28). The Wisconsin Card Sorting Test (WCST) is a widely used neuropsychological assessment that reflects the function of executive decision-making areas in the frontal lobe and is employed to evaluate cognitive flexibility in both healthy and clinical populations (29). Studies have shown that individuals with schizophrenia perform worse on the WCST compared to the general population, indicating the presence of cognitive dysfunction related to executive functions in these patients (30, 31). Given that both fNIRS and the WCST provide complementary measures of cognitive function, we will utilize them to evaluate the cognitive functions of patients with anhedonia, aiming to explore whether improvements in anhedonia are associated with cognitive function.

This study aims to: (1) evaluate the efficacy and safety of DMPFC-iTBS for treating anhedonia in schizophrenia; (2) examine the relationship between anhedonia severity, prefrontal cortical activity, and cognitive performance; and (3) explore potential neurobiological mechanisms with the help of fNIRS.

## 2 Methods

### 2.1 Study design

This double-blind, randomized controlled trial will recruit 82 patients with schizophrenia and have a score of ≥12 on the Snaith–Hamilton Pleasure Scale (SHAPS) from the Zigong Affiliated Hospital of Southwest Medical University. Participants will be randomly assigned in a 1:1 ratio to receive either active or sham iTBS, with identical parameters and procedures aside from the stimulation condition. This study is conducted in compliance with the World Medical Association’s Code of Ethics (Declaration of Helsinki). All participants will be fully informed of the study protocol and will provide written informed consent.

**Figure 1:**
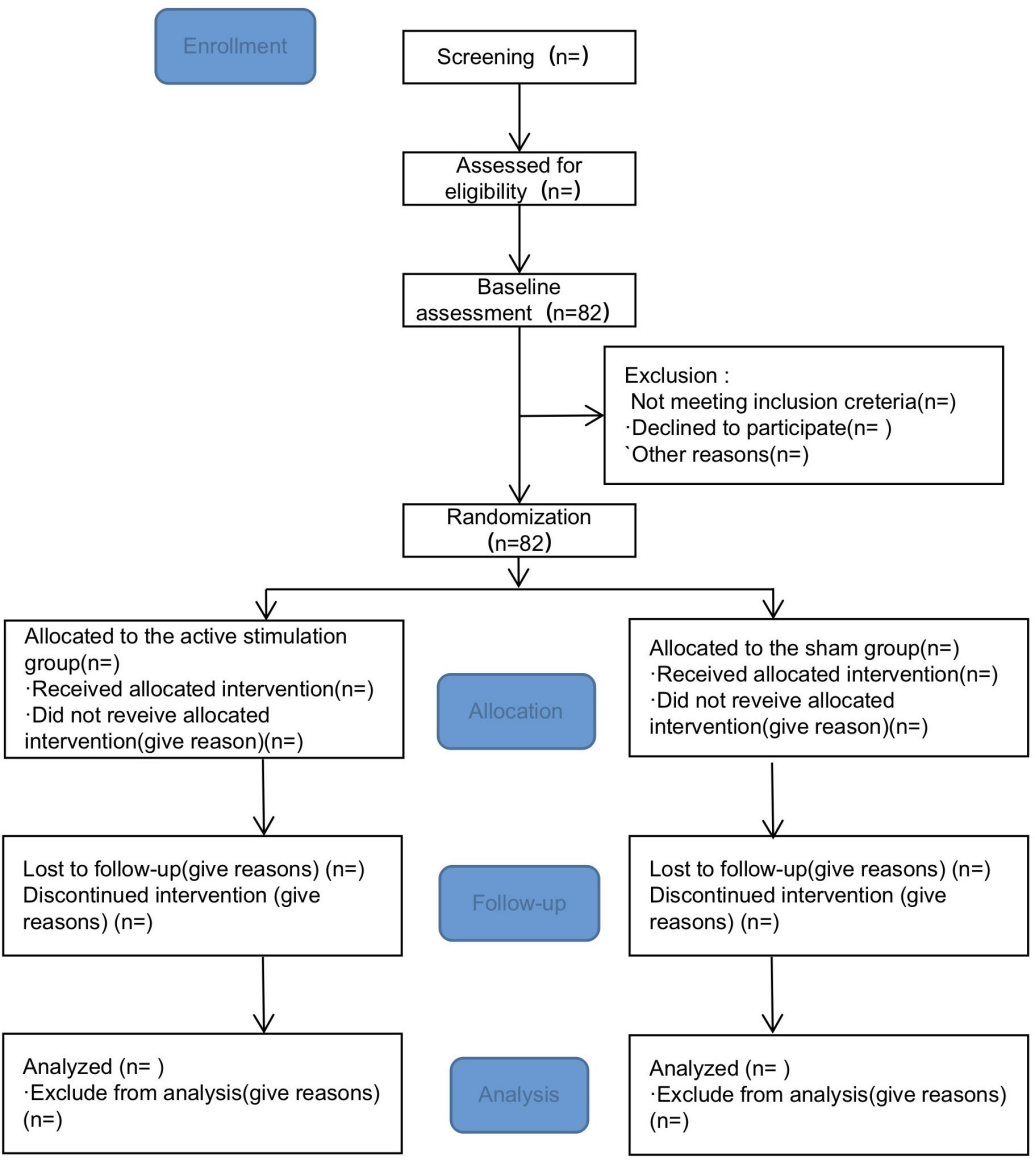
Overview of study procedure.

Specifically, Individuals expressing interest in the program will first complete a screening questionnaire covering basic demographics and the SHAPS. Those who meet the preliminary inclusion criteria will be invited by their physician to participate. After providing written informed consent, eligible participants will undergo a comprehensive baseline assessment. This assessment includes the collection of clinical data, clinical scale evaluations, WCST, and an fNIRS scan. Following the baseline assessment, participants will be randomly assigned to either the active iTBS group or the sham stimulation group based on the number of randomization in the sealed envelope. The study comprises two phases: a treatment period (15 days) and a follow-up period (4 weeks). All participants in both groups will be evaluated using the SHAPS and the Scale for the Assessment of Negative Symptoms (SANS), and will undergo fNIRS scanning at three time points: before treatment (T0), immediately after treatment (T1), and 4 weeks after treatment completion (T2) (**Figure 1**).

### 2.2 Participants

Inclusion Criteria: 1) Aged 18–55 years; right-handed; 2) Individuals diagnosed with schizophrenia according to DSM-5; 3)Normal or corrected-to-normal vision; 4) On a stable medication regimen for at least two months prior to enrollment; 5) Naïve to the WCST; 6) Participants agreed to forgo any new psychotherapy for the duration of the trial.

Exclusion Criteria: 1) Current diagnosis of substance use disorder, personality disorder, depression, bipolar disorder, schizoaffective disorder or intellectual disability; 2) Color blindness; 3) Major cardiovascular, hepatic, or renal disease; 4) Pregnancy or lactation; 5) Any intracranial metal implants or objects; 6) With a history of electroconvulsive therapy (ECT) within the preceding three months; 7) with a history of seizure. The complete criteria are detailed in **Table 1**.

**Table 1:** Inclusion and exclusion.

Inclusion criteria	Exclusion criteria
1.Individuals diagnosed with schizophrenia according to DSM-5 2.Score of ≥12 on the Snaith-Hamilton Pleasure Scale (SHAPS). 3.Aged between 18 and 55 years. 4.Medication regimen stable for at least two months. 5. Right-handed. 6. Have normal or corrected-to-normal vision and hearing 7.No prior experience with the Wisconsin Card Sorting Test (WCST). 8.No concurrent psychotherapy.	1.Current substance abuse or dependence, depression, bipolar disorder. 2.Diagnosed with personality disorders. 3.Presence of intellectual disability. 4.Color blindness. 5.Diagnosed with severe cardiovascular, hepatic, or renal disease. 6.Currently pregnant or lactation. 7.Any intracranial metal implants or objects. 8.History of modified electroconvulsive therapy (MECT) within the preceding three months. 9. with a history of seizure.

### 2.3 Randomization and blinding

Participants were assigned via simple randomization using a computer-generated random sequence. Simple random allocation was performed by generating uniform random numbers within the [0,1] interval via the RAND() function in Microsoft Excel. These random numbers were ranked in ascending order, with the first half assigned to the active stimulation group and the second half to the sham group.

An independent statistician, who had no other involvement in the trial, generated and maintained the random sequence. To ensure allocation concealment, assignments were placed in sequentially numbered, opaque, sealed envelopes, which were stored securely. The envelopes were provided to the rTMS technician. The technician was aware of the group assignments but was not involved in outcome assessments or any other aspect of the study, thereby protecting the blinding of investigators and participants. The study investigators and participants will remain blinded to the treatment conditions until all data have been collected.

### 2.4 Interventions

The DMPFC treatment target was defined as 25% of the nasion-inion distance in the middle-line of the scalp (32). Stimulation of bilateral hemisphere was accomplished by orienting the coil perpendicular to midline, with current flow directed toward the hemisphere to be stimulated. We use a Classic Magnetic Stimulator YRD CCY-IIB (Yiruide, Wuhan, China).

**Figure 2:**
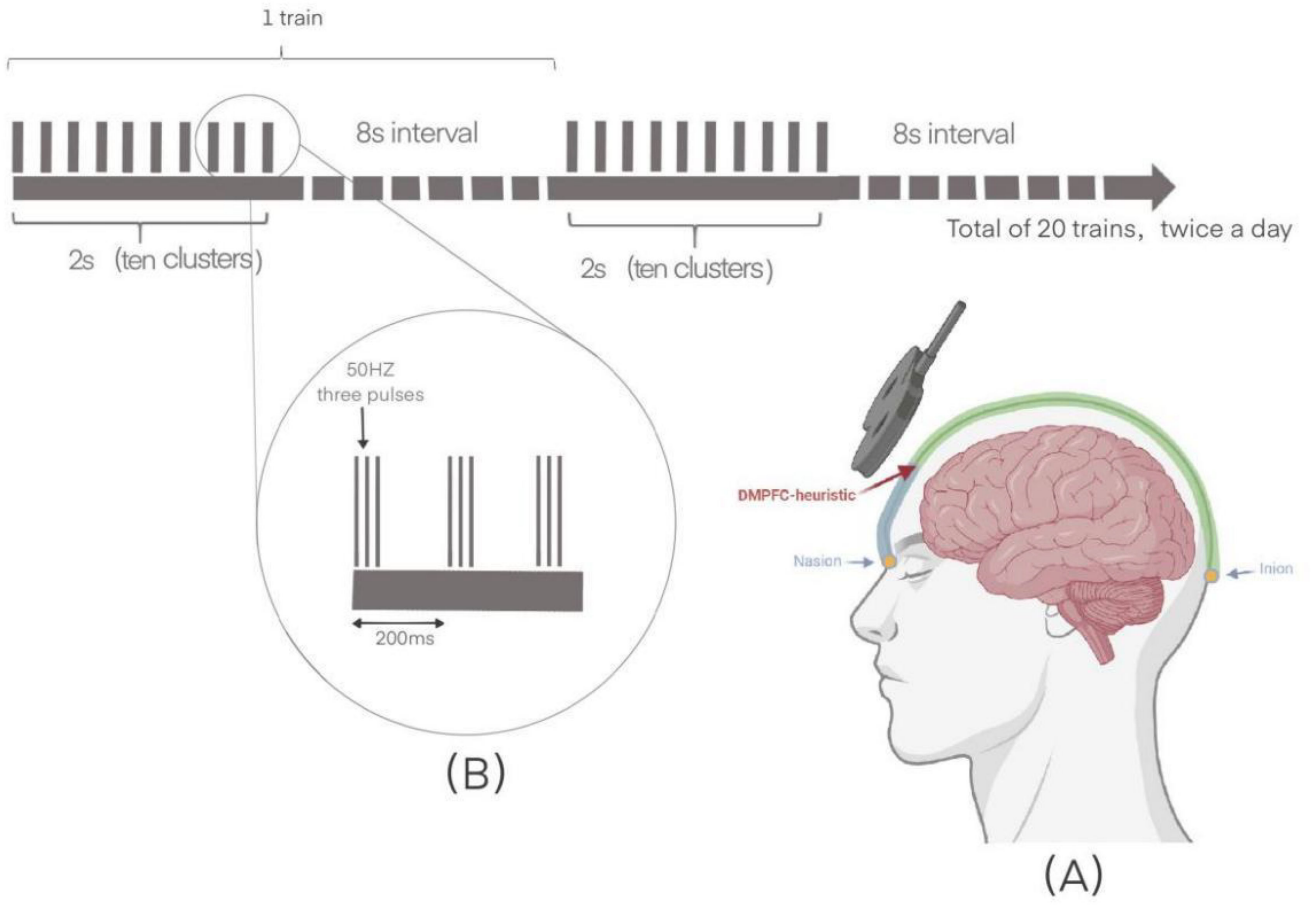
The schematic diagram of the iTBS. **(A)** The dorsomedial prefrontal cortex surface localization: 25% of the nasion-inion distance. **(B)** The left and right dorsomedial prefrontal cortex each received 20 trains of stimulation. Each train consisted of 2 seconds of stimulation followed by an 8-second interval. The stimulation included 10 clusters at 5Hz, with each cluster containing 3 biphasic pulses at 50Hz, totaling 1200 pulses per session. This treatment was administered twice daily for 15 days.

In the active stimulation group, magnetic stimulation is performed at 90% of the resting motor threshold, with 20 trains of the left and right DMPFC. Preferential stimulation of each hemisphere was accomplished by lateral coil orientation (33). The rTMS target was first randomized to the left or right DMPFC, then switched to the opposite side. (i.e., 40trains, including 20 right side over left side, or 20 left side over right side). The left or right DMPFC were counterbalanced between subjects. Each train consisted of two seconds of stimulation with an eight-second interval. This stimulation consisted of ten 5 HZ clusters with three 50 HZ biphasic pulses in each pulse for a total of 1200 pulses per session. To accelerate the treatment and promote plasticity, a second identical treatment was performed after a 15-minute break (34). The treatment consisted of 30 sessions, administered twice daily over a 15-day period. Sham stimulation was provided to the control group with the coil tilted 90°to the scalp, which produced noise and vibratory sensations similar to real stimuli. And all participants continued their original medication therapy (**Figure 2**).

### 2.5 Outcome measurements

The main outcome measure is the change in scores on the SHAPS. Secondary outcome indicators: 1) The change in the SANS; 2) The change in the Side Effects Scale (SEPS); 3) The change in the Temporal Experience of Pleasure Scale (TEPS); 4) The oxygenated hemoglobin (oxy-Hb) and the deoxyhemoglobin (deoxy-Hb) in the probe and channel at the region of interest-prefrontal cortex, under the near-infrared functional brain imaging task; 5) Results of the Wisconsin Card Test: number of correct, number of incorrect, number of persistent errors, number of nonsustained errors, total number of classifications, and number of items needed to complete the first classification; 6) the Adverse Events (AEs) (**Table 2**).

**Table 2:** Outline of study assessments and timelines.

Assessment tool	Baseline	Post-interventions (Day16)	Follow-up (Day44)
Demographic information	✓		
SHAPS	✓	✓	✓
TEPS	✓	✓	✓
SANS	✓	✓	✓
fNIR	✓	✓	✓
WCST	✓	✓	✓
AEs		✓	✓

SHAPS, the Snaith-Hamilton Pleasure Scale; TEPS, the Scale for the Assessment of Negative Symptoms; TEPS, the change in the Temporal Experience of Pleasure Scale; fNIR, functional near-infrared spectroscopy; WCST, the Wisconsin Card Test; AEs, Adverse Events.

#### 2.6.1 fNIRS acquisition

The fNIRS data will be collected at three time points: baseline, post-intervention, and follow-up. Measurements will be obtained using an fNIRS device (BS-3000L, Wuhan Union Medical Technology Co., Wuhan, China) to quantify regional oxygen saturation. All fNIRS assessments will be conducted in a quiet, controlled environment. Throughout the entire process, all participants will be instructed to remain quiet and minimize head movements during the measurements. The fNIRS assessment will be conducted during the performance of a Verbal Fluency Task (VFT) which is used to measure changes in blood oxygen concentrations. Based on the probe and channel layout, the region of interest (ROI) will be defined as the prefrontal cortex, and the oxy-Hb and deoxy-Hb concentrations will be quantified for each channel.

**Figure 3:**
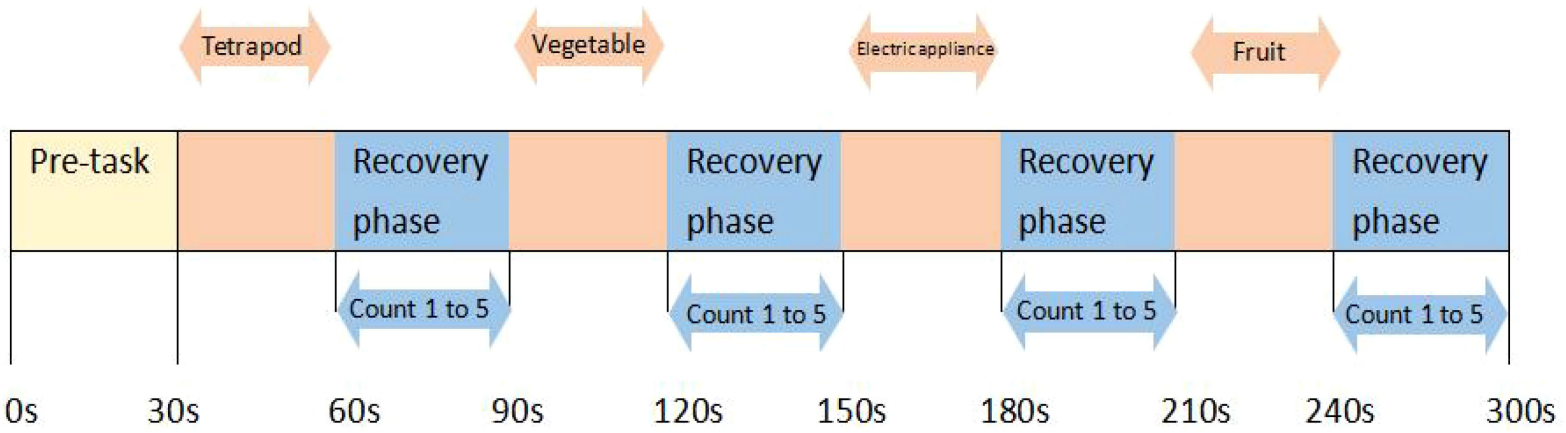
The procedure of the fNIRS. A 30-second pre-scan is conducted before the task begins to stabilize the data. During the task, participants are required to list as many items as possible from four categories (tetrapod, vegetable, electric appliance, fruit) in random order. During the rest period, participants need to repeatedly count from 1 to 5 following the computer screen.

Briefly, at the beginning of the task, there is also a 30-second pre-scan to stabilize the data. During the execution of the task, the participant needs to list as many items as possible that belong to a particular category (categories include four-legged animals, vegetables, household appliances, and fruits), and the order in which each category appears is randomized. During the recovery period of the task, participants will be asked to repeat the counting from 1 to 5 following the computer screen to exclude the effect of head movements during talking on the data during the task period. The task periods and all other recovery periods are 30 seconds long, except for the final recovery period, which is 60 seconds. The specific words uttered by the participants in each block will be recorded and judged to meet the criteria. The average number of correct items spoken in each of the four blocks will be calculated and taken as the participant’s performance on the VFT task (**Figure 3**).

#### 2.6.2 WCST data collection

**Figure 4:**
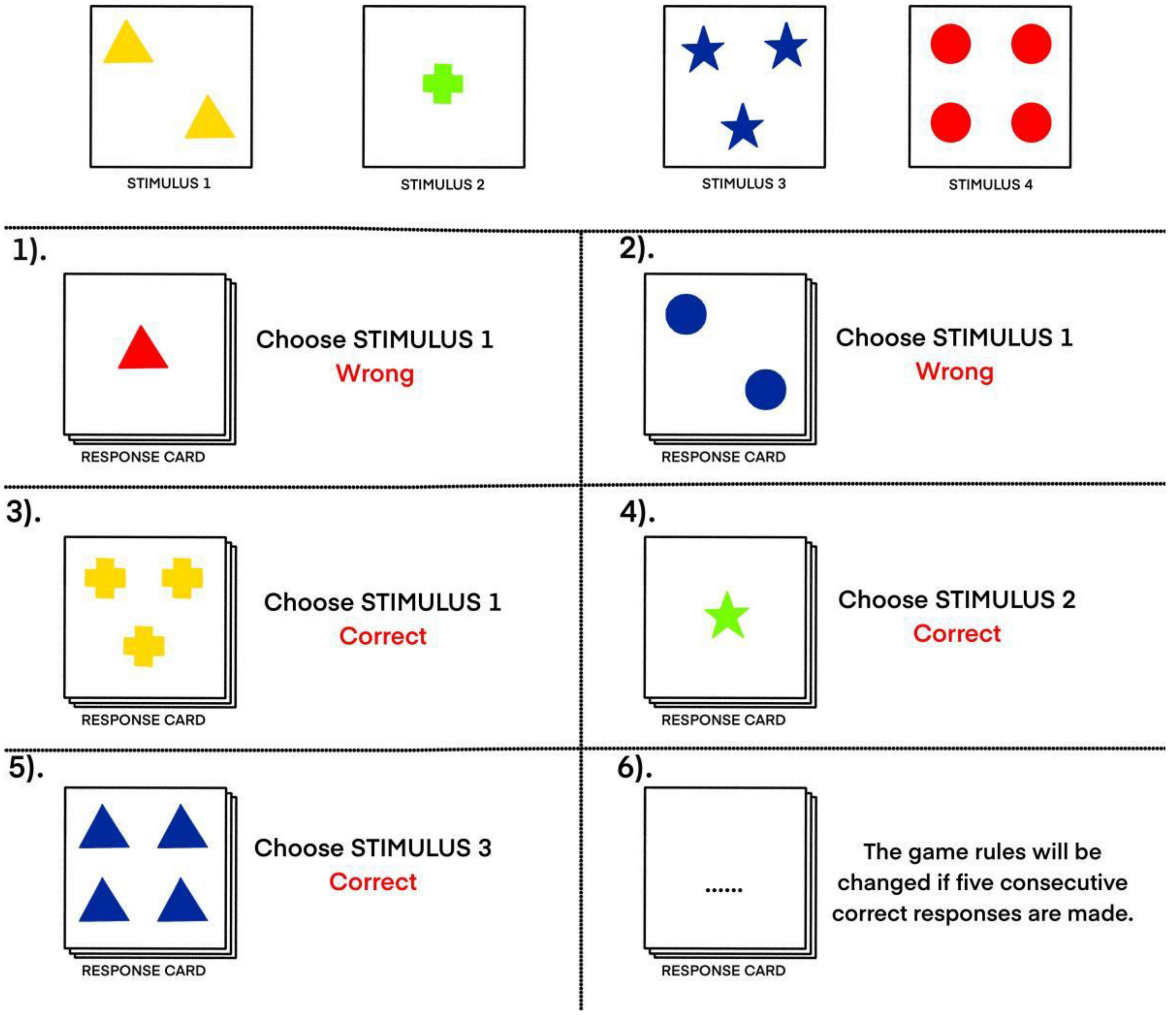
An example of the WSCT display. Participants will complete a card-matching task in which they must discover a hidden rule (based on color, shape, or number) by comparing a response card to four stimulus cards. All cards vary across these three dimensions. Feedback is provided after each attempt, and the rule changes after five correct responses in a row to test cognitive flexibility.

Participants will be presented with four stimulus cards that will vary across three dimensions: color (red, green, blue, yellow), shape (circle, star, triangle, cross), and number of shapes (one, two, three, four). A response card also will be presented, which will vary similarly along these three dimensions. The participant’s task will be to match the response card to one of the stimulus cards based on an unknown matching rule related to one of the three dimensions (e.g., color, shape, or number). Participants will not initially be informed of the correct dimension and will be required to deduce the rule through trial and error, receiving feedback after each attempt. To increase complexity and assess cognitive flexibility, the matching rule will change after every five consecutive correct responses, requiring participants to continuously adapt their strategies. The following performance indicators will be recorded: number of correct responses, number of total errors, number of perseverative errors (repeating a previously correct rule despite feedback), number of non-perseverative errors, total number of categories completed, and number of trials required to complete the first category (**Figure 4**).

### 2.7 Statistical analysis

#### 2.7.1 Sample size

This study focuses on the differences in outcomes due to the two populations at the three time points, and the statistical methodology lends itself to the use of repeated measures ANOVA. The dependent variable was set as the measurement outcome. The independent variables were between-groups for the rTMS group and sham rTMS group, and within-groups for the three time points of pre-treatment (T0), post-treatment (T1), and 4 weeks post-treatment (T2). The effect size used to calculate the effect size was 0.25, the significance level alpha was 0.05, the statistical test efficacy was 0.8, and the non-spherical corrected moderate deviation from the spherical hypothesis value was 0.75. We will need a sample size of 66 participants. If the maximum allowable dropout rate is 20%, the total sample size needed for this study would be 82 participants. The sample size calculation was carried out using G*Power version 3.1.9.7_143.

#### 2.7.2 Statistical considerations

Differences in demographic characteristics and clinical variables between the two groups will be compared using t-test for continuous variables and χ^2^ test for categorical variables. The primary and secondary outcomes will be analyzed using linear mixed-effects models, applied on an intention-to-treat basis. In addition, Pearson or Spearman correlations will be used to analyze the correlation of anhedonia with prefrontal activity and cognitive performance. All statistical analyses were performed using SPSS (Version 24.0; IBM Corp., Armonk, NY, USA).

To assess the integrity of blinding, participants were asked at the conclusion of the treatment period to guess their group assignment (active or sham). The distribution of guesses between the two groups was compared using a χ² test. A correct guess rate no better than chance (50%) in both groups would be considered evidence of successful blinding.

#### 2.7.3 Security analysis

The safety aspects of this intervention will need to be taken into account, so we will record all adverse events and negative consequences. The safety analysis will include descriptive statistics for all randomized subjects.

## 3 Discussion

The design of this protocol is based on the following key evidence. Recent studies have shown that rTMS targeting the DMPFC can alleviate anhedonia in depression by improving connectivity within the reward circuitry (15, 35). Anhedonia is a symptom of apathy in schizophrenia, but it is also a core symptom of depression. Some perspectives suggest that the overlap between anhedonia and apathy in schizophrenia can be explained by specific deficits in anticipatory pleasure, while consummatory pleasure experience remains relatively intact (36, 37). The mechanisms underlying anhedonia differ between schizophrenia and depression. As reviewed by Bégue et al., anhedonia in schizophrenia is associated with abnormalities in the prefrontal-striatal network, particularly involving the ventral striatum (VS), ventromedial prefrontal cortex (VMPFC), dorsolateral prefrontal cortex (DLPFC), and anterior cingulate cortex (ACC). Although DMPFC-iTBS has been explored in depression, its application in schizophrenia remains underexplored, representing an innovative strategy for targeting the complex network underlying anhedonia in this disorder (38). Furthermore, previous clinical studies have demonstrated the efficacy of DMPFC-targeted rTMS in schizophrenia with negative symptoms (39). We anticipate that the rTMS intervention in this study will lead to significant improvements in anhedonia among schizophrenia.

Anhedonia is associated with multiple reward processing components, including reward evaluation, anticipation, decision-making, and motivation (40). It is thought to potentially arise from dysfunction in the mesolimbic dopamine pathway and its terminal regions (e.g., striatum, amygdala, and prefrontal cortex) (41). It is noteworthy that these brain regions may also be involved in motivational processing (42), suggesting a possible shared pathological basis between anhedonia and amotivation in schizophrenia. The DMPFC, as a key node within the mesolimbic dopaminergic system (14), may be a promising target for rTMS to ameliorate anhedonia in schizophrenia.

To elucidate the potential neurobiological mechanisms of action, our trial collected cognition-related data, such as prefrontal cortex oxyhemoglobin levels measured via fNIRS. If analyses reveal correlations between improvements in anhedonia and these cognitive metrics, it could offer clues for further explaining the intervention mechanisms of rTMS.

Several limitations of this study should be acknowledged. Firstly, although every effort was made to maintain participant blinding, it is possible that some individuals may have inferred their intervention assignment during the trial based on their prior rTMS experience. Secondly, the study included only a 4-week follow-up period, resulting in a relatively short overall duration and limiting the evaluation of long-term efficacy. Finally, the use of fNIRS to assess brain function in this study is constrained by its limited resolution and penetration depth, which hampers precise localization of neural activity. Future research could incorporate multimodal imaging techniques, such as magnetic resonance imaging, to improve anatomical accuracy and provide deeper mechanistic insights.

In summary, if our study demonstrates that high-frequency iTBS targeting the DMPFC is a safe and effective approach for alleviating anhedonia in schizophrenia, it could offer a novel avenue for treating this symptom in affected patients.

### 3.1 Test status

The study is currently ongoing. Recruitment started in January 2025 and will end in December 2025.
